# The Ability of NEWS2 to Detect Sepsis in Adult Patients With Positive Blood Cultures

**DOI:** 10.1111/apm.70129

**Published:** 2025-12-28

**Authors:** Karolina Liljedahl Prytz, Anders Magnuson, Martin Sundqvist, Lisa Kurland, Jan Källman

**Affiliations:** ^1^ Department of Infectious Diseases, Faculty of Medicine and Health Örebro University Örebro Sweden; ^2^ Clinical Epidemiology and Biostatistics, School of Medical Sciences, Faculty of Medicine and Health Örebro University Örebro Sweden; ^3^ Department of Laboratory Medicine, Clinical Microbiology, Faculty of Medicine and Health Örebro University Örebro Sweden; ^4^ Department of Emergency Medicine, Faculty of Medicine and Health Örebro University Örebro Sweden

**Keywords:** BSI, community‐acquired, emergency department, NEWS2, sepsis, SOFA

## Abstract

Blood stream infections are associated with high mortality and morbidity. NEWS2 is a quick scoring system including bedside measurable vital signs. This study aimed to investigate the ability of NEWS2 ≥ 5p to identify sepsis, per Sepsis‐3 criteria, among adult patients with community‐acquired infection and positive blood cultures. It also explored if NEWS2 ≥ 5p could indicate infection etiology based on bacterial species in blood culture. This retrospective study included 555 patients with positive blood cultures. 425 of 555 (76.6%) patients had sepsis. The sensitivity of NEWS2 ≥ 5p for detecting sepsis was 86.6% and was not statistically associated with infection etiology. Patients with *S. pneumoniae* had a higher median NEWS2 score than those with other bacterial species. The 28‐day mortality rate was 12.1%, and the sensitivity of NEWS2 ≥ 5p for detecting 28‐day mortality was 91.0%. NEWS2 ≥ 5p was detected in a high proportion of sepsis cases among patients with blood stream infections, independent of bacterial species, and is a quick tool for identifying high sepsis likelihood in the emergency department.

## 1 Background

Blood stream infections (BSIs) are associated with high mortality and significant morbidity [1, 2, 3]. The visible clinical signs of sepsis can vary from patient to patient depending on age [4, 5], sex [6], bacterial agent causing the infection [7, 8, 9], comorbidity [10], and time from first symptoms [11]. Studies have also shown that hospital employees (nurses and physicians) interpret symptoms differently [12, 13, 14].

Hospitals use different scoring systems to objectively monitor sick patients. The Sequential Organ Failure Assessment score (SOFA score) was widely accepted as the fundamental criterion of Sepsis‐3 in 2016. It was designed as a research tool to assess the acute morbidity of critical illness in groups of patients with sepsis so that they could be categorized on the basis of their risk of death [15]. It is constructed of three clinical variables and three blood tests reflecting organ function [16]. Some of the variables used in the SOFA scoring system can be difficult to assess within the 1‐h time constraint or are not routinely taken in the prehospital or emergency department setting. The SOFA score requires extensive clinical information, and some of that information is frequently missing [15].

The National Early Warning Score (NEWS) was developed in 2012 and revised in 2017 (NEWS2) with support from the National Health Service (NHS), United Kingdom. NEWS2 is quick, easy to perform, does not require any blood sampling and includes vital signs measurable at the bedside. Although NEWS2 is also recommended as a risk stratification and identification tool in patients with suspected sepsis, it has not been developed specifically for sepsis identification [17].

Prior investigations have demonstrated that an aggregated NEWS2 score of ≥ 5 represents an optimal threshold of sensitivity and specificity for indicating potential clinical deterioration in patients with severe infectious illnesses both in hospital settings and in prehospital [18] and admission contexts [19, 20]. Previous studies have investigated the sensitivity of the NEWS2 score for detecting sepsis independent of blood culture results. The correlations between vital signs and bacterial species found in blood cultures have not been sufficiently investigated.

An early signal related to the etiology, possibly indicated by the NEWS2 score, could serve as a valuable tool to guide antibiotic treatment, facilitate rapid initiation of source control, and supportive care [21, 22, 23, 24], particularly in patients with infections caused by common bacteria associated with high mortality and morbidity rates, such as *Escherichia coli* [9], *Staphylococcus aureus* [8], and *Streptococcus pneumoniae* [7]. This approach could subsequently contribute to expedited recovery, reduced morbidity, and improved patient outcomes [22, 24].

Compared with blood culture‐negative sepsis patients, patients with sepsis and positive blood cultures have increased severity of illness, increased mortality, and a deteriorated standardized mortality ratio [25, 26, 27]. Research has demonstrated that patients with septic shock present higher blood culture positivity rates [28].

The primary aim of this study was to investigate the ability of a NEWS2 ≥ 5p to identify sepsis, which is defined in accordance with the Sepsis‐3 criteria, among adult patients with community‐acquired infection and positive blood cultures. Furthermore, the aim of this study was to explore the possibility of NEWS2 ≥ 5p to provide an indication of the etiology of the infection based on Gram staining and the bacterial species found in blood culture.

## 2 Materials and Methods

### 2.1 Study Design and Setting

This retrospective study included patients attending the emergency department (ED) at Örebro University Hospital, Örebro, Sweden, and two nearby university‐affiliated hospitals. The three EDs in the region had approximately 107,000 adult patient visits annually during the study period. The cohort of adult patients with positive blood cultures due to community‐acquired infections has been described previously, with a focus on antibiotic treatment [29]. This study is a new analysis of the same cohort. Since it is a population‐based observational study, no sample size calculation was performed.

### 2.2 Ethical Approval

Ethical approval was obtained from the Uppsala Ethical Review Board, Sweden, in December 2013 (EPN 201/451; O63‐13). All methods were performed in accordance with relevant guidelines and regulations. Since this was a retrospective study with no impact on treatment choices or outcomes, a waiver of informed consent was granted by the Ethical Review Board.

### 2.3 Participants

All consecutive patients with positive blood cultures obtained between January 1, 2011, and December 31, 2012, in the Örebro County region were considered for inclusion. Eligible patients were those admitted directly from home, without ongoing antibiotic or chemotherapy treatment, and not receiving dialysis. Patients younger than 18 years, those with nosocomial infections, or with positive blood cultures regarded as contaminants (for definition see [29]) were excluded.

### 2.4 Patient Characteristics and Laboratory Results

All Information regarding patient characteristics, comorbidities, and laboratory results at admission was retrieved from the patients’ medical records. Baseline characteristics such as age, sex, and isolated pathogens were documented, and comorbidities were calculated according to the Charlson Comorbidity Index (CCI) [10]. The laboratory results included the platelet blood count, serum creatinine level, and serum bilirubin level.

### 2.5 Definitions

Disease severity was defined by the Sepsis‐3 criteria and according to the SOFA score categorized as either non‐sepsis, sepsis, or septic shock [16]. Sepsis was defined as organ dysfunction characterized by an acute increase of ≥ 2 points in the total SOFA score, septic shock as sepsis plus s‐lactate > 2 mmol/L, and vasopressor treatment [16, 30]. Previously accepted formulas were used to estimate the mean arterial pressure (MAP) and PaO_2_/FIO_2_ [30, 31, 32].

### 2.6 NEWS2

A positive NEWS2 score indicating sepsis was defined as a NEWS2 score ≥ 5p and negative otherwise. The maximum congregated score is 20 points, and the threshold to alert for sepsis is ≥ 5 points [17].

### 2.7 Data Collection

The SOFA score and NEWS2 score were not routinely used to identify sepsis in 2011 and 2012. Instead, another triage system was used, Medical Emergency Triage and Treatment System (METTS) [33]. In this system, vital signs are recorded by medical staff upon arrival at the ED. The documentation of vital signs was used to calculate the NEWS2 and SOFA scores in the current study.

### 2.8 Microbiological Data

During the study period, blood cultures were performed according to standard procedures. Two sets of blood cultures, one aerobic bottle and one anaerobic bottle in each set, were routinely collected. Blood culture flasks were incubated with the BACTEC FX system (Becton Dickinson, Franklin Lakes, NJ, USA). Standard methods in the laboratory at the time of the study were used to determine the genus and species of the microorganisms. The results of blood cultures were obtained from the Department of Clinical Microbiology at Örebro University Hospital. Organisms commonly recovered from the environment or the skin (mainly coagulase‐negative staphylococci) were assessed as contaminants, except when clinical findings (i.e., the results of cultures from other body sites or two or more positive sets) indicated a high probability of bloodstream infection [29].

### 2.9 Data Analysis

SPSS version 22 and STATA release number 17 were used for the statistical analysis. Continuous variables were summarized as the median and interquartile range (IQR). Categorical variables are reported as counts and percentages.

Mann–Whitney test was used to compare continuous variables, and chi‐squared test or Fisher's exact test was used for categorical variables between patients with complete versus missing SOFA score.

The Kruskal–Wallis test and Mann–Whitney test were used to compare the NEWS2 score between different blood culture results. ROC (receiver operative curve) was used to show the sensitivity and specificity for different cut‐off values of NEWS2 score in relation to SOFA ≥ 2 score (the gold standard for detecting sepsis according to Sepsis‐3). The measures of diagnostic accuracy, between a NEWS2 ≥ 5p and a SOFA score ≥ 2p were calculated and supplemented with 95% confidence intervals (CIs), using binomial distribution. Poisson regression, with robust standard errors, was used to compare the diagnostic accuracy measurements between groups based on gram‐staining results and between bacterial species ( *Escherichia coli* , *Staphylococcus aureus* , and *Streptococcus pneumoniae* ). As a sensitivity analysis, the specificity and NPV were performed, with missing SOFA score set to negative, this to adjust for the possible effects of excluding patients with missing SOFA scores, which only could affect the specificity, not the sensitivity. Statistical significance was set at *p* < 0.05.

## 3 Results

### 3.1 Participants

**FIGURE 1:**
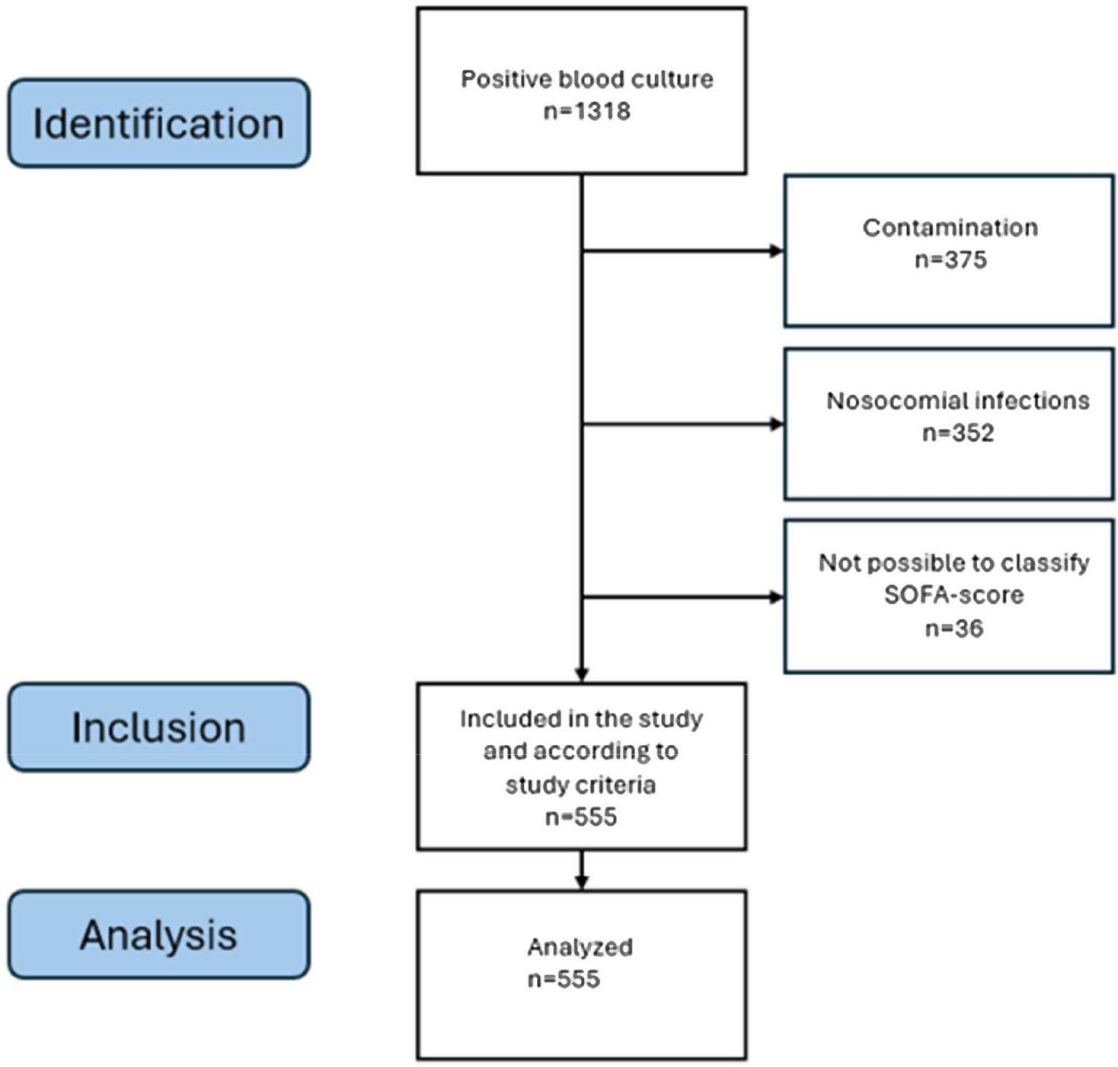
Flowchart for inclusion and exclusion.

A total of 1318 patients with positive blood cultures were identified. Patients with nosocomial infections (*n* = 352) or blood cultures positive for bacteria regarded as contaminants (*n* = 375) were excluded. The SOFA scores were incomplete for 129 patients. The missing variables were the coagulation score (*n* = 28), liver score (*n* = 126), and kidney score (*n* = 2). Among these 129 patients, 93 reached ≥ 2 points despite an incomplete SOFA score and were therefore included in the final analysis. The remaining patients (*n* = 36) were excluded from this study. After assessment of the exclusion criteria (see above), 555 patients were included for further analyses (Figure 1).

The median age was 76 years, with 51.7% males. 115 patients had chronic kidney disease as a diagnosis in their medical record and 111 had both chronic disease and raised creatinine at baseline. Distribution of CCI and bacterial are shown in Table 1. The most prevalent pathogen was *Escherichia coli* (35.1%), followed by *Staphylococcus aureus* (15.7%), and *Streptococcus pneumoniae* (13.0%).

**TABLE 1:** Baseline characteristics.

	Included (*n* = 555)
Age, median (IQR)	76 (65–85)
Women, *n* (%)	268 (48.3%)
Men, *n* (%)	287 (51.7%)
Septic shock, *n* (%)	79 (14.2)
CCI, *n* (%)	
0	160 (28.8)
1–2	226 (40.7)
3+	169 (30.5)
Gram‐positive, *n* (%)	250 (45.0)
*S. aureus* , *n* (%)	87 (15.7)
*S. pneumoniae* , *n* (%)	72 (13.0)
Gram‐negative, *n* (%)	305 (55.0)
*E. coli* , *n* (%)	195 (35.1)
Other findings in blood culture^a^, *n* (%)	201 (36.2)

Abbreviation: CCI, Charlson Comorbidity Index.

^a^ See Table S1 for details.

### 3.2 The Ability of a NEWS2 Score ≥ 5p to Detect Sepsis (SOFA Score ≥ 2p)

The ROC showed the sensitivity and the specificity for different cut‐off values of NEWS2 score in relation to SOFA ≥ 2 score with an AUC of 0.77. A cut‐off at 5 points showed the best combination of sensitivity and specificity (Figure S1).

A total of 425 patients (76.6%, 95% CI 72.8–80.0) presented with sepsis (SOFA score ≥ 2p), and 431 (77.6%, 95% CI 73.9–81.1) presented with a NEWS2 score ≥ 5p. The sensitivity of a NEWS2 score ≥ 5p for detecting sepsis independent of etiology was 86.6% (95% CI 83.0–89.7). The corresponding sensitivity for Gram‐positive infections was 88.9%, and that for Gram‐negative infections was 84.5% (RR 1.05; 95% CI 0.98–1.13). The sensitivities for specific pathogens were 85.4% among patients with *Escherichia coli* , 85.1% for *Staphylococcus aureus* , and 91.8% for *Streptococcus pneumoniae* (Table 2).

**TABLE 2:** Diagnostic accuracy of NEWS2 ≥ 5p classification in relation to SOFA score ≥ 2p in total and by bacterial species.

	*n*	SOFA ≥ 2	NEWS2 ≥ 5^a^ sensitivity	Unadjusted		Adjusted^b^		SOFA < 2	NEWS2 < 5^a^ specificity	Unadjusted		Adjusted^b^	
		no.	no., % (95% CI)	RR (95% CI)	*p*	RR (95% CI)	*p*	no.	no., % (95% CI)	RR (95% CI)	*p*	RR (95% CI)	*p*
All	555	425	368, 86.6% (83.0–89.7)					130	67, 51.5% (42.6–60.4)				
Gram‐positive	250	199	177, 88.9% (83.7–92.9)	1.05 (0.98–1.13)	0.18	1.06 (0.98–1.15)	0.10	51	29, 56.9% (42.2–70.7)	1.18 (0.85–1.65)	0.32	1.30 (0.92–1.86)	0.14
Gram‐negative	305	226	191, 84.5% (79.1–89.0)	Reference		Reference		79	38, 48.1% (36.7–59.6)	Reference		Reference	
*E. coli*	195	144	123, 85.4% (78.6–90.7)	Reference		Reference		51	27, 52.9% (38.4–67.1)	Reference		Reference	
*S. aureus*	87	67	57, 85.1% (74.3–92.6)	0.99 (0.88–1.12)	0.95	1.00 (0.89–1.13)	0.96	20	12, 60.0% (36.0–80.9)	1.13 (0.73–1.77)	0.58	1.51 (0.90–2.54)	0.12
*S. pneumoniae*	72	61	56, 91.8% (81.9–97.3)	1.07 (0.97–1.19)	0.16	1.12 (1.01–1.26)	0.036	11	6, 54.6% (23.4–83.3)	1.03 (0.56–1.88)	0.92	1.01 (0.55–1.85)	0.97
Other findings in blood culture^c^	201	153	132, 86.3% (80.0–91.3)	1.01 (0.92–1.11)	0.83	1.02 (0.93–1.12)	0.63	48	22, 45.8% (31.4–60.8)	0.86 (0.58–1.30)	0.48	0.91 (0.60–1.38)	0.66

	*n*	NEWS2 ≥ 5^a^	SOFA ≥ 2 PPV	Unadjusted		Adjusted^b^		NEWS2 < 5^a^	SOFA < 2 NPV	Unadjusted		Adjusted^b^	
		no.	no., % (95% CI)	RR (95% CI)	*p*	RR (95% CI)	*p*	no.	no., % (95% CI)	RR (95% CI)	*p*	RR (95% CI)	*p*
All	555	431	368, 85.4% (81.6–88.6)					124	67, 54.0% (44.8–63.0)				
Gram‐positive	250	199	177, 88.9% (83.7–92.9)	1.08 (1.00–1.17)	0.049	1.09 (1.01–1.18)	0.023	51	29, 56.9% (42.2–70.7)	1.09 (0.79–1.51)	0.60	1.14 (0.81–1.61)	0.44
Gram‐negative	305	232	191, 82.3% (76.8–87.0)	Reference		Reference		73	38, 52.0% (40.0–63.9)	Reference		Reference	
*E. coli*	195	147	123, 83.7% (76.7–89.3)	Reference		Reference		48	27, 56.2% (41.2–70.5)	Reference		Reference	
*S. aureus*	87	65	57, 87.7% (77.2–94.5)	1.05 (0.93–1.18)	0.43	1.05 (0.93–1.18)	0.45	22	12, 54.6% (32.2–75.6)	0.97 (0.61–1.53)	0.90	1.06 (0.63–1.80)	0.82
*S. pneumoniae*	72	61	56, 91.8% (81.9–97.3)	1.10 (0.99–1.22)	0.080	1.17 (1.04–1.30)	0.006	11	6, 54.6% (23.4–83.3)	0.97 (0.53–1.76)	0.92	0.99 (0.54–1.80)	0.96
Other findings in blood culture^c^	201	158	132, 83.5% (76.8–89.0)	1.00 (0.90–1.10)	0.98	1.03 (0.93–1.13)	0.57	43	22, 51.2% (35.5–66.7)	0.91 (0.62–1.34)	0.63	0.87 (0.57–1.31)	0.50

^a^ Positive NEWS2‐score was determined if the sum of all individual points were ≥ 5 points.

^b^ Adjusted for age in 5 years intervals (< 65, 65–69, 70–74, 75–79, 80–84, 85–89, ≥ 90), sex and CCI (0, 1–2, ≥ 3). RR, relative risk ratio; CI, confidence interval.

^c^ See Table S1 for details.

The specificity of a NEWS2 score ≥ 5p for the prediction of sepsis in blood culture‐positive patients was 51.5% (95% CI 42.6–60.4) (Table 2).

In the sensitivity analyses (when patients with missing SOFA score were set as negative), the specificity was 53.0% (95% CI 45.1–60.8) (Table S2).

### 3.3 Distribution of the SOFA Score and NEWS2 Score in Relation to Bacterial Species

**FIGURE 2:**
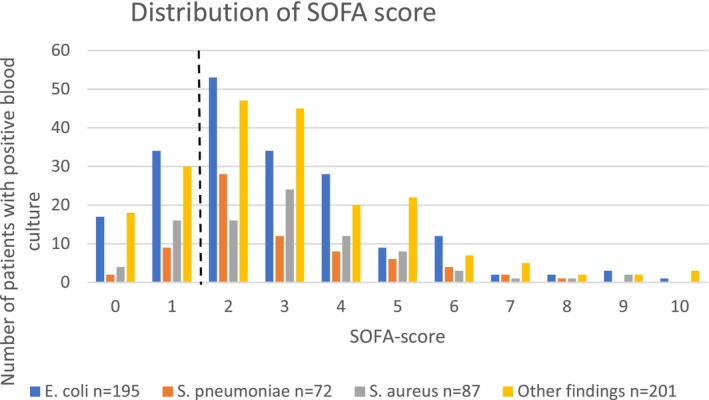
Distribution of SOFA scores. Distribution of SOFA scores (*n* = 555). Median 3 (IQR 2–4). *E. coli* (blue) median 2 (IQR 1–4), *S. pneumoniae* (orange) median 2 (IQR 2–4), *S. aureus* (gray) median 3 (IQR 2–4), and other findings (yellow) median 3 (IQR 2–4). For further information about other findings in blood culture, please see Table S1. The SOFA score to define sepsis (≥ 2 points) is marked with a dashed line.

**FIGURE 3:**
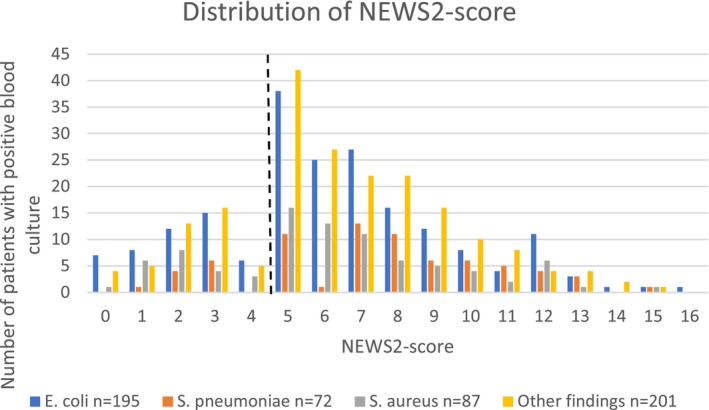
Distribution of the NEWS2 score. Distribution of the NEWS2 score (*n* = 555), median score of 6, minimum score of 0, maximum score of 16, and IQR of 5–8. *E. coli* (blue) median 6, IQR 5–8; *S. pneumoniae* (orange) median 7.5, IQR 5–10; *S. aureus* (gray) median 6, IQR 4–8; and other findings (yellow) median 6, IQR 5–8. The NEWS2 score for identifying sepsis (≥ 5 points) is marked with a dashed line.

No significant differences were observed among the causative pathogens with respect to the number of patients with a NEWS2 ≥ 5p. However, as the total score per patient is dependent on various variables, a secondary analysis was conducted to describe the distribution of score values in relation to the pathogen for both the SOFA score and the NEWS2. The distribution of SOFA scores was similar irrespective of the etiology of the infection (Figure 2). In contrast, examination of the NEWS2 score distribution (Figure 3) revealed a statistically significant difference in the distribution of pathogens causing infection (*p* = 0.014, Kruskal–Wallis test). Patients infected with *S. pneumoniae* exhibited a higher overall NEWS2 score than did those in each of the other subgroups ( *S. pneumoniae* vs. *E. coli* , *p* = 0.002; *S. aureus* , *p* = 0.007; and other bacteria, *p* = 0.007; Mann–Whitney test).

### 3.4 Mortality Risk Predicted by SOFA Score > 2p and NEWS2 ≥ 5p

Sixty‐seven out of 555 patients died within 28 days (12.1%, 95% CI 9.5–15.1). A SOFA score ≥ 2p identified 63 (94.0%, 95% CI 85.4–98.3) of these patients, and a NEWS2 score ≥ 5p identified 61 (91.0%, 95% CI 81.5–96.6) (ns). Fifty‐nine of the 67 patients who died within 28 days were defined as having sepsis according to both scoring systems. However, the SOFA score identified four additional patients, whereas a NEWS2 score ≥ 5p identified two additional patients. No significant differences in mortality were observed between the bacterial species.

## 4 Discussion

This study investigated adult patients with community‐acquired infections and positive blood culture results. Among the 555 patients in this cohort identified in the ED based on positive blood cultures, 425 (76.6%) presented with sepsis.

Overall, a NEWS2 score ≥ 5p demonstrated the ability to detect most sepsis cases with a sensitivity of 86.6% and a specificity of 51.5% but was not statistically associated with the etiology of the infection. The cut‐off threshold of NEWS2 ≥ 5p has been validated in prior research [18, 19, 20], and our study did not identify a superior cut‐off level. However, patients with *S. pneumoniae* infection presented, as a group, with higher NEWS2 scores than did those with other bacterial species. The 28‐day mortality rate in patients with a NEWS2 score ≥ 5p was 12.1%, which is consistent with previous studies [34, 35, 36]. No significant differences in mortality were observed among the bacterial species; however, the statistical power was limited due to the small number of deaths.

The sensitivity of a NEWS2 score ≥ 5p for the prediction of sepsis in this study was marginally greater than that reported by McGrath et al. [37], who reported a sensitivity of 62.1%; Yu et al. [38], who reported a sensitivity of 75.8%; and Usman et al. (74.3%) [39]. However, none of the abovementioned studies investigated a strict blood culture‐positive population or exclusively community‐acquired infections, which may have affected the results. Positive blood culture was established as an inclusion criterion because previous studies reported increased mortality in this cohort [1, 2, 3], and our aim was to investigate the potential prediction of the etiology upon presentation to the ED.

NEWS2 is a valid scoring system for the early detection of sepsis in the ED [37, 38, 39]. In this study, stratified for patients with positive blood cultures and community‐acquired infections, we confirmed that the NEWS2 reached an even higher sensitivity for detecting sepsis, thus indicating the potential for an early signal regarding which patients may require prompt antibiotic treatment and intensive care to mitigate the increased risk of mortality. However, a NEWS2 < 5p cannot definitively exclude sepsis. Prior studies have highlighted the issue of sepsis overdiagnosis within emergency departments [40, 41]. Both SOFA score and NEWS2 score have high sensitivity but lower specificity [16, 18] but since sepsis is one of the most fatal medical emergencies and also a time critical condition, this speaks for creating identification tools with a high sensitivity. During the initial hour of emergency care, clinicians may exhibit a tendency to diagnose sepsis prematurely, even when the patient's condition is less severe or not genuinely septic. This inclination occurs prior to the availability of laboratory results and examinations that could confirm whether the condition is indeed an infection or attributable to other factors affecting the scores [40]. After identifying a patient with suspected sepsis, this assessment needs to be scrutinized by a specialist/experienced physician, which are shown to demonstrate a high specificity [42, 43]. Clar et al. proposed an amplified NEWS2, that is, NEWS2 in combination with PaCO2 ≤ 35 mmHg, lactate ≥ 2.0, or glucose ≥ 190 mg/dL, to increase the sensitivity for sepsis detection [44]. This represents one of several potential approaches for future warning scores to become more sensitive in identifying sepsis. However, this aspect was not investigated in this study and will directly limit the utility of the score, as these modified versions, similar to the SOFA, necessitate blood‐based analysis.

Notably, patients whose blood cultures were positive for *S. pneumoniae* presented higher NEWS2 scores than patients with other bacterial species did, and very few patients positive for *S. pneumoniae* received a score of 4 or 6. The SOFA score did not differ on the basis of bacterial etiology and generally displayed a more uniform distribution of score values. This observation may be attributed to the more rapid onset of fever and respiratory distress in patients with *S. pneumoniae* , resulting in higher NEWS2 scores [7], in conjunction with the requirement for supplemental oxygen to maintain their recommended saturation [17]. Concurrently, studies and clinical experience have indicated that patients with Gram‐negative sepsis experience greater impacts on blood pressure and circulation due to lipopolysaccharide (LPS) [9, 45], which could also influence the NEWS2 score. Research has shown that infections caused by Gram‐negative bacteria are associated with increased levels of inflammatory markers and more frequently fulfill the criteria for sepsis [46, 47, 48]. In this study and in the study by Sorensen et al. [49], a tendency towards the opposite was observed, as Gram‐positive bacteria more frequently fulfilled the sepsis criteria.

Rapid detection of etiological pathogens is a priority. It facilitates the appropriate selection of antibiotic treatment, prompt initiation of source control, and supportive care. This approach may subsequently contribute to expedited recovery, reduced morbidity, and improved patient outcomes [22, 24]. The potential for swift assessment of patients with diverse symptoms in the ED is advantageous, and a scoring system yielding results within < 5 min is desirable. The current gold standard, the SOFA score, is more applicable in the intensive care unit (ICU) than in the ED [44], as three of the variables in the SOFA score necessitate blood sampling and analysis, which requires time, resources, and laboratory facilities. We showed that, irrespective of the etiology, a NEWS2 ≥ 5p largely indicated a sepsis episode. The use of the NEWS as an early alternative to the SOFA score could enhance the initial care of patients in the ED by reducing the time to physician consultation, antibiotic administration, and fluid treatment.

## 5 Strengths and Limitations

This study was a retrospective study and thus has a potential risk of selection bias. However, the utilization of a population‐based sample of patients with positive blood cultures obtained in the ED strengthened the results, and only 36 patients lacked the necessary information for inclusion in the study.

An additional limitation was the low statistical power owing to the limited number of patients when comparing sensitivities between bacterial subgroups. Although the study population was collected several years ago, the pathogens studied remain significant causes of sepsis, and most variables could be retrieved; thus, we believe that the results are still valid today. The study was confined to patients with positive blood cultures; consequently, no conclusions could be drawn regarding the performance of the NEWS2 in the cohort with negative blood cultures. However, this study design may have led to the selection of more severely ill patients, potentially resulting in an overestimation of the performance of NEWS2.

## 6 Conclusions

In summary, a NEWS2 score ≥ 5p demonstrated the ability to detect most sepsis cases. The NEWS2 score was significantly higher in patients infected with *S. pneumoniae* than in those infected with other bacterial species. Furthermore, a NEWS2 ≥ 5p exhibited high (91.0%) sensitivity for the prediction of mortality within 28 days. Based on these findings, we confirmed that a NEWS2 score ≥ 5p serves as a valuable initial screening tool for identifying a high likelihood of sepsis in the ED and that a NEWS2 score ≥ 5p should prompt further investigation. However, a NEWS2 < 5p does not definitively rule out sepsis, and additional diagnostic measures are necessary to identify these patients.

## Funding

The study was financed by grants from the Swedish state under the agreement between the Swedish government and the Örebro county council, the so‐called ALF agreement.

## Ethics Statement

Ethical approval was obtained from the Uppsala Ethical Review Board Sweden in December 2013 (EPN 201/451, O63‐13). All methods were conducted in accordance with relevant guidelines and regulations. Since this study is a retrospective study with no impact on treatment choices or outcomes, a waiver of informed consent was granted by the Ethical Review Board of Uppsala, Sweden.

## Conflicts of Interest

The authors declare no conflicts of interest.

## Supporting information


**Figure S1:** The ROC‐curve comparing sensitivity and specificity for different NEWS2 score cut‐off levels in agreement to SOFA ≥ 2p.

**Table S1:** Description of the distribution of bacterial species (count and percentage).

**Table S2:** Sensitivity analysis of the specificity and NPV of NEWS2 ≥ 5p classification in relation to SOFA score ≥ 2p including the 36 patients with incomplete SOFA scores as negative.

## Data Availability

The data that support the findings of this study are available on request from the corresponding author.
