# Supplementary material for: The Ability of NEWS2 to Detect Sepsis in Adult Patients With Positive Blood Cultures
Source: APMIS. 2025 Dec 28;133(12):e70129. doi: 10.1111/apm.70129 (PMC12745187; doi:10.1111/apm.70129)
Supplement: Supplementary file 1 — Figure S1: The ROC‐curve comparing sensitivity and specificity for different NEWS2 score cut‐off levels in agreement to SOFA ≥ 2p. [file APM-133-0-s001.docx]

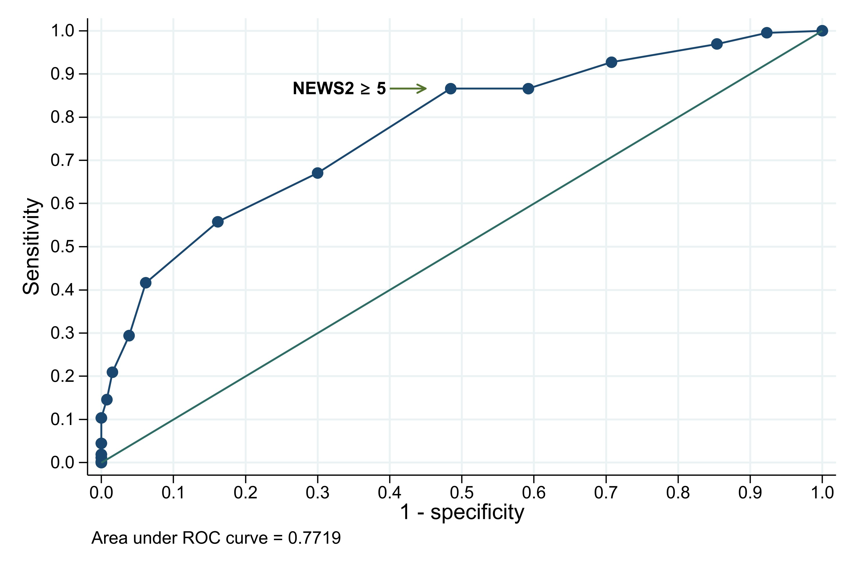


Figure S1. The ROC-curve comparing sensitivity and specificity for different NEWS2 score cut-off levels in agreement to SOFA ≥2p.
