# Supplementary material for: The Ability of NEWS2 to Detect Sepsis in Adult Patients With Positive Blood Cultures
Source: APMIS. 2025 Dec 28;133(12):e70129. doi: 10.1111/apm.70129 (PMC12745187; doi:10.1111/apm.70129)
Supplement: Supplementary file 2 — Table S1: Description of the distribution of bacterial species (count and percentage). [file APM-133-0-s003.docx]

Table S1. Description of the distribution of bacterial species (count and percentage)

| Bacterial species | Valid  (n=555) | Percent (%) |
| --- | --- | --- |
| *Escherichia coli* | 195 | 35.1 |
| *Staphylococcus aureus* | 87 | 15.7 |
| Streptococcus pneumoniae | 72 | 13.0 |
| *Klebsiella pneumoniae* | 35 | 6.3 |
| Viridans streptococci | 27 | 4.9 |
| *Pseudomonas aeruginosa* | 14 | 2.5 |
| Beta-hemolytic streptococcus Group G | 12 | 2.2 |
| *Enterococcus faecalis* | 11 | 2.0 |
| Beta-hemolytic streptococcus Group B | 10 | 1.8 |
| *Streptococcus pyogenes* | 9 | 1.6 |
| *Bacteroides fragilis* | 9 | 1.6 |
| Coagulase negative staphylococci | 9 | 1.6 |
| *Proteus mirabilis* | 9 | 1.6 |
| *Enterobacter* spp. | 8 | 1.4 |
| *Salmonella* spp. | 7 | 1.3 |
| *Haemophilus influenzae* | 6 | 1.1 |
| *Pasteurella multocida* | 6 | 1.1 |
| *Clostridium* spp. | 4 | 0.7 |
| *Fusobacterium necrophorum* | 3 | 0.5 |
| Beta-hemolytic streptococcus Group C | 3 | 0.5 |
| Anaerobic gram positive cocci | 2 | 0.3 |
| *Campylobacter jejunii* | 2 | 0.3 |
| *Klebsiella oxytoca* | 2 | 0.3 |
| *Serratia marcescens* | 2 | 0.3 |
| *Staphylococcus saprophyticus* | 2 | 0.3 |
| *Acinetobacter baumanii* | 1 | 0.2 |
| *Aerococcus* spp | 1 | 0.2 |
| *Capnocytophaga canimorsus* | 1 | 0.2 |
| *Francisella tularensis* | 1 | 0.2 |
| *Laktobacillus* spp. | 1 | 0.2 |
| *Moraxella catarrhalis* | 1 | 0.2 |
| *Neisseria meningitidis* serogroup Y | 1 | 0.2 |
| *Raoultella* spp. | 1 | 0.2 |
| *Streptobacillus moniliformis* | 1 | 0.2 |
