# Supplementary material for: The Ability of NEWS2 to Detect Sepsis in Adult Patients With Positive Blood Cultures
Source: APMIS. 2025 Dec 28;133(12):e70129. doi: 10.1111/apm.70129 (PMC12745187; doi:10.1111/apm.70129)
Supplement: Supplementary file 3 — Table S2: Sensitivity analysis of the specificity and NPV of NEWS2 ≥ 5p classification in relation to SOFA score ≥ 2p including the 36 patients with incomplete SOFA scores as negative. [file APM-133-0-s002.docx]

Table S2. Sensitivity analysis of the specificity and NPV of NEWS2≥5p classification in relation to SOFA score ≥2p including the 36 patients with incomplete SOFA scores as negative.

|  |  | **SOFA<2** | **NEWS2≥5p**  **specificity** | **Unadjusted** | | **Adjusted ^a^** | |
| --- | --- | --- | --- | --- | --- | --- | --- |
|  | n | no. | no., % (95% CI) | RR (95% CI) | *p* | RR (95% CI) | *p* |
| All | 591 | 166 | 88, 53.0% (45.1-60.8) |  |  |  |  |
| Gram positive | 266 | 67 | 36, 53.7% (41.1-66.0) | 1.02 (0.76-1.37) | *0.88* | 1.12 (0.82-1.53) | *0.47* |
| Gram negative | 325 | 99 | 52, 52.5% (42.2-62.7) | Reference |  | Reference |  |
| *E. coli* | 212 | 68 | 39, 57.4% (44.8-69.3) | Reference |  | Reference |  |
| *S. aureus* | 93 | 26 | 15, 57.7% (36.9-76.6) | 1.00 (0.68-1.48) | *0.98* | 1.24 (0.79-1.94) | *0.35* |
| *S. pneumoniae* | 76 | 15 | 7, 46.7% (21.3-73.4) | 0.81 (0.45-1.45) | *0.49* | 0.82 (0.46-1.49) | *0.52* |
| Other findings in blood culture ^c^ | 210 | 57 | 27, 47.4% (34.0-61.0) | 0.82 (0.59-1.16) | *0.27* | 0.91 (0.63-1.30) | *0.61* |
|  |  | **NEWS2≥5** | **SOFA<2**  **NPV** | **Unadjusted** | | **Adjusted ^a^** | |
|  | n | no. | no., % (95% CI) | RR (95% CI) | *p* | RR (95% CI) | *p* |
| All | 591 | 145 | 88, 60.7% (52.2-68.7) |  |  |  |  |
| Gram positive | 266 | 58 | 36, 62.1% (48.4-74.5) | 1.04 (0.80-1.35) | *0.78* | 1.07 (0.81-1.40) | *0.64* |
| Gram negative | 325 | 87 | 52, 59.8% (48.7-70.1) | Reference |  | Reference |  |
| *E. coli* | 212 | 60 | 39, 65.0% (51.6-76.9) | Reference |  | Reference |  |
| *S. aureus* | 93 | 25 | 15, 60.0% (38.7-78.9) | 0.92 (0.64-1.34) | *0.67* | 0.95 (0.62-1.44) | *0.80* |
| *S. pneumoniae* | 76 | 12 | 7, 58.3% (27.7-84.8) | 0.90 (0.54-1.50) | *0.68* | 0.90 (0.54-1.50) | *0.68* |
| Other findings in blood culture ^b^ | 210 | 48 | 27, 56.2% (41.1-70.5) | 0.86 (0.63-1.18) | *0.36* | 0.82 (0.59-1.15) | *0.26* |

^a^Adjusted for age in 5 years intervals (<65, 65-69, 70-74, 75-79, 80-84, 85-89, ≥90), sex and CCI (0, 1-2, ≥3).

CI: confidence interval. NPV: Negative predicted value ^b^See Supplement Table S1 for details
